# Temperature Controls Crystalline Iron Oxide Utilization by Microbial Communities in Methanic Ferruginous Marine Sediment Incubations

**DOI:** 10.3389/fmicb.2018.02574

**Published:** 2018-10-30

**Authors:** David A. Aromokeye, Tim Richter-Heitmann, Oluwatobi E. Oni, Ajinkya Kulkarni, Xiuran Yin, Sabine Kasten, Michael W. Friedrich

**Affiliations:** ^1^Microbial Ecophysiology Group, Faculty of Biology/Chemistry, University of Bremen, Bremen, Germany; ^2^MARUM, Center for Marine Environmental Sciences, University of Bremen, Bremen, Germany; ^3^International Max Planck Research School for Marine Microbiology, Max-Planck-Institute for Marine Microbiology, Bremen, Germany; ^4^Alfred Wegener Institute Helmholtz Centre for Polar and Marine Research, Bremerhaven, Germany; ^5^Faculty of Geosciences, University of Bremen, Bremen, Germany

**Keywords:** DIET, temperature control, marine sediment, iron reduction, methanogenesis, microbial community analysis

## Abstract

Microorganisms can use crystalline iron minerals for iron reduction linked to organic matter degradation or as conduits for direct interspecies electron transfer (mDIET) to syntrophic partners, e.g., methanogens. The environmental conditions that lead either to reduction or conduit use are so far unknown. We investigated microbial community shifts and interactions with crystalline iron minerals (hematite and magnetite) in methanic ferruginous marine sediment incubations during organic matter (glucose) degradation at varying temperatures. Iron reduction rates increased with decreasing temperature from 30°C to 4°C. Both hematite and magnetite facilitated iron reduction at 4°C, demonstrating that microorganisms in the methanic zone of marine sediments can reduce crystalline iron oxides under psychrophilic conditions. Methanogenesis occurred, however, at higher rates with increasing temperature. At 30°C, both hematite and magnetite accelerated methanogenesis onset and maximum process rates. At lower temperatures (10°C and 4°C), hematite could still facilitate methanogenesis but magnetite served more as an electron acceptor for iron reduction than as a conduit. Different temperatures selected for different key microorganisms: at 30°C, members of genus *Orenia*, Halobacteroidaceae, at 10°C, *Photobacterium* and the order Clostridiales, and at 4°C *Photobacterium* and *Psychromonas* were enriched. Members of the order Desulfuromonadales harboring known dissimilatory iron reducers were also enriched at all temperatures. Our results show that crystalline iron oxides predominant in some natural environments can facilitate electron transfer between microbial communities at psychrophilic temperatures. Furthermore, temperature has a critical role in determining the pathway of crystalline iron oxide utilization in marine sediment shifting from conduction at 30°C to predominantly iron reduction at lower temperatures.

## Introduction

Iron oxide minerals are ubiquitous in natural environments (Straub et al., 2001; Kappler and Straub, 2005; Braunschweig et al., 2013) and exist chemically as amorphous, poorly crystalline or crystalline phases (Cornell and Schwertmann, 2003). Microbial reduction of amorphous and poorly crystalline iron oxide phases is thermodynamically more favorable than using crystalline phases (Weber et al., 2006); therefore, microorganisms preferentially utilize less crystalline phases for dissimilatory and fermentation-linked iron reduction (Munch and Ottow, 1980; Munch and Ottow, 1983; Lovley and Phillips, 1986, 1987; Lovley, 1991). There is growing evidence that the more crystalline iron oxide mineral phases may nevertheless support microbial metabolism either as (I) terminal electron acceptors during oxidation of complex organic matter or fermentation end products such as acetate and H_2_ (Lentini et al., 2012; Hori et al., 2015) or as (II) conduits for electron transfer between microorganisms living in syntrophic association (Kato et al., 2012; Liu et al., 2015).

Several incubation studies with marine sediments have previously demonstrated the feasibility of microbial reduction of amorphous iron(III) and poorly crystalline phases like ferrihydrite under psychrophilic (Zhang et al., 1999; Stapleton et al., 2005; Roh et al., 2006; Vandieken et al., 2006), mesophilic (Roden and Lovley, 1993) and thermophilic conditions (Kashefi and Lovley, 2003; Kashefi et al., 2008; Manzella et al., 2013). Microbial reduction of crystalline iron oxides such as goethite (α-FeOOH), hematite (α-Fe_2_O_3_) and magnetite [Fe(II)Fe(III)_2_O_4_] was previously demonstrated under mesophilic (Roden and Zachara, 1996; Lentini et al., 2012; Hori et al., 2015) but not under psychrophilic conditions. Meanwhile, elevated concentrations of dissolved Fe^2+^ have been detected in the methanic zone of some sub-seafloor sediments bearing appreciable amounts of these crystalline mineral phases (e.g., Hensen et al., 2003; Riedinger et al., 2005; März et al., 2008; Riedinger et al., 2014; Oni et al., 2015a; Egger et al., 2017). Therefore, microbial reduction of crystalline iron oxides could be a prime metabolic process in these sediment layers. Despite the large body of work on iron reduction at varying temperature conditions and in various environments, knowledge of the diversity of microorganisms involved in crystalline iron mineral reduction in cold marine sediments is still limited.

Mineral-mediated electron transfer between metabolically dependent microbes is particularly important in methanogenic environments such as flooded rice field soils (Kato et al., 2012; Zhou et al., 2014). Despite mineral-meditated direct interspecies electron transfer (mDIET) being central to the functioning of methane-producing microbial communities, the environmental factors that govern these interactions are yet to be studied (Shrestha and Rotaru, 2014). In contrast to other environments such as rice field soils (Kato et al., 2012; Zhou et al., 2014; Li et al., 2015; Zhuang et al., 2015) freshwater (Jiang et al., 2013; Zhang and Lu, 2016) and coastal sediments (Rotaru et al., 2018), microbial communities capable of mDIET in methanic marine sediments are not known.

Although it is established that microorganisms from different environments can reduce crystalline iron minerals or utilize them as conduits to facilitate electron transfer to syntrophic partners, controls of these interactions under different environmental conditions are not known yet. Here, we posit that temperature controls the mode of crystalline iron minerals utilization, either reduction, conduction or both. To address this hypothesis, we specifically studied (1) the role of crystalline iron minerals during microbial community shifts in marine sediment incubations under varying temperature conditions, (2) the potential for reduction of crystalline iron minerals in sub-seafloor sediments at different temperatures and (3) identified microbial populations that can interact with crystalline iron minerals in marine sediments under varying temperature regimes.

## Materials and Methods

### Sampling Site

The Helgoland Mud Area is a highly depositional site of fine-grained mud located in the German Bight, North Sea. The depositional history and geochemical profiles of this site were previously described (Hebbeln et al., 2003; Oni et al., 2015a). Samples were collected using a gravity corer (5 m core length) during RV HEINCKE research expedition HE 443 (54° 05.23′ N; 007° 58.04′ E) in May 2015. The core HE443/077-1 was stored at 4°C on board, transported to the laboratory within a few days of core retrieval, and sliced into 25-cm sections. Each 25-cm section was stored at 4°C in 2.6-L jars, under a headspace of N_2_ (99.999% purity, Linde, Germany).

### Incubation Experiments

Anoxic 50-ml slurry incubations were prepared in 120-ml serum vials with sediment from 416 to 441 and 441 to 466 cm depths and anoxic sulfate-free artificial sea water (ASW; composition [L^-1^]: 26.4 g NaCl, 11.2 g MgCl_2_⋅6H_2_O, 1.5 g CaCl_2_⋅2H_2_O and 0.7 g KCl) at a ratio of 1:3 (w/v) under a headspace of N_2_. Incubations (*n* = 9) were supplemented by adding ∼1,020 μmoles of iron oxides (hematite or magnetite; LanXess, Germany) and 68 μmoles of glucose as electron donor. Control incubations (“glucose only,” *n* = 9) were supplemented with 68 μmoles glucose only (Supplementary Table S1). Control sediment slurry incubations containing iron oxides only were considered not necessary as previous incubation studies with subsurface sediments from the Helgoland mud area demonstrated that endogenous organic matter is not reactive enough to stimulate iron reduction and methanogenesis within tolerable laboratory incubation times (∼200 days; Oni, 2015). The sediments of the Helgoland Mud Area are rich in different phases of iron minerals (up to 0.8 wt %; Oni et al., 2015a). Hence, adding a carbon source (glucose) to the sediment even without adding iron oxides stimulated microbial iron reduction in the sediments. The potential for reduction of amended hematite (HG) or magnetite (MG) was evaluated by comparing the amount of Fe^2+^ formed in crystalline iron-treated incubations to those of the “glucose only” control (G). Triplicates of each treatment set were incubated statically in the dark at 4°C, 10°C, or 30°C. Triplicate supplementary incubations were set up at 30°C, for testing the effect of the inhibitor 2-bromoethanesulfonate (BES) on methanogenesis and iron reduction in the presence of crystalline iron oxides. This was achieved by adding ∼15 mM BES to freshly prepared triplicate slurries (“glucose only,” magnetite and glucose, hematite and glucose). Another set-up in duplicates was done for all treatments across all temperatures to investigate the effects of pH during glucose fermentation (Supplementary Table S2).

### Analytical Methods

All incubations were first sampled after approximately 12 h at respective temperatures, and designated as “day 0.” One milliliter of slurry was collected under anoxic conditions in 1.5 ml reaction tubes pre-flushed with N_2_. HCl extractable Fe(II) was determined for each sample first by mixing 100 μl of slurry from each sample with 100 μl 0.5 M HCl. The mixture was subsequently incubated at room temperature for 24 h. Afterward, the supernatant was collected by centrifugation (15,300 × *g*, 5 min) followed by spectrophotometric determination of Fe(II). However, we observed high amounts of HCl extractable Fe(II) at day 0 that likely originated from precipitated iron carbonate (e.g., siderite) and sulfur compounds (e.g., FeS) within sediment samples. Given the large background of sediment indigenous Fe(II) compounds, it was not possible to determine accurately the amount of freshly formed Fe(II) over the course of the incubation, which precipitates rather rapidly (Supplementary Figure S1). Therefore, the aqueous Fe^2+^ measured was used as a proxy for evaluating iron reduction kinetics. This was done by centrifugation of freshly collected anoxic slurry and directly adding 100 μl of supernatant from each centrifuged sample (15,300 × *g*, 5 min) to ferrozine reagent following Viollier et al. (2000). The rest of the slurry was stored at -20°C and subsequently used for DNA extraction where required.

Methane concentrations in incubation headspace samples (100 μl) were monitored over time using a gas chromatograph (GC) (Shimadzu GC-2014, Tokyo, Japan) coupled to a methanizer (nickel reactor, CP 11952, Agilent, Germany). GC was equipped with a flame ionization detector and a packed column (Porapak Q, 2 m × 1/8″; inner diameter 2 mm, mesh range: 80/100; Agilent, Waldbronn, Germany). H_2_ served both as carrier gas (99.999% purity; 500 kPa, 30 ml min^-1^ flow rate) and combustion gas (40 kPa). Compressed air (50 kPa) was used for combustion, while make-up gas was N_2_ (500 kPa). Temperature conditions were as follows; detector (200°C), injector (120°C), column (70°C), and methanizer (350°C). Chromatographic data was recorded using a Peak Simple data system (model 2002, SRI, Bad Honnef, Germany). Methane amounts formed in headspace were calculated using the ideal gas law with incubation temperature as variable. Methanogenesis rates were systematically evaluated for each time-point by dividing the methane concentration change by the time elapsed between two successive time-points measured per replicate (Δ[CH_4_]^∗^Δt^-1^).

### Nucleic Acid Extraction

One milliliter of slurry from individual incubations, at specific time points, was used for nucleic acid extraction following a modified protocol from Lueders et al. (2004). Nucleic acids were precipitated from aqueous supernatant by adding two volumes of 30% polyethylene glycol (PEG-6000) followed by centrifugation (15,300 × *g*, 90 min at 4°C). Pellets were washed twice with 500 μl 70% ethanol (15,300 × *g*, 5 min at 4°C) followed by elution in 50 μl diethylpyrocarbonate (DEPC) treated water (Carl Roth, Germany). Nucleic acid concentrations were measured with NanoDrop 1000 spectrophotometer (Peqlab Biotechnologie, Erlangen, Germany). For further processing, day 0 samples from the “glucose only” incubations were taken as day 0 for all incubations. All extracted nucleic acids were stored at -20°C until use.

### Amplification of 16S rRNA Genes and Illumina Hiseq Sequencing

Illumina amplicon sequencing PCR was prepared using primers targeting 16S rRNA genes of either Bacteria or Archaea. Bacteria targeting primers used were Bac515F (5′-GTGYCAGCMGCCGCGGTAA-3′; Parada et al., 2016) and Bac805R (5′-GACTACHVGGGTATCTAATCC-3′; Herlemann et al., 2011). Archaea targeting primers were Arc519F (5′-CAGCMGCCGCGGTAA-3′; Ovreås et al., 1997) and Arc806R (GGACTACVSGGGTATCTAAT; Takai and Horikoshi, 2000). Each primer had in addition a unique barcode sequences (8 bp; Hamady et al., 2008) that facilitated multiplexing of several samples in one sequence library.

PCR reaction mix (50 μl) contained 1 x KAPA HiFi buffer, 0.3 mM dNTP mix, 0.25 U KAPA HiFi DNA polymerase (KAPA Biosystems, Germany), 1.5 μM each of forward and reverse primer pairs, and 2 μl of 10x diluted DNA template from each sample. Thermal cycling conditions include initial denaturation at 95°C for 5 min, final denaturation at 98°C for 20 s, 20 s of annealing at 60°C, extension at 72°C for 20 s and final elongation at 72°C for 1 min. A total of 28 PCR cycles were run. PCR products were screened by gel electrophoresis before purification using Monarch^®^ PCR and DNA purification kit (New England Biolabs, Germany). PCR products were quantified using Quant-iT PicoGreen dsDNA assay kit (Thermo Fisher Scientific, United States). Based on the measured quantities by PicoGreen, a library of samples was constructed using equimolar amounts from each amplicon. Amplicon library was sequenced at GATC GmbH (Konstanz, Germany) using the Illumina 2^∗^150 base pairs HiSeq 4000 Platform.

### Sequence Analysis

Sequence analysis was performed on the QIIME 1.8.0 platform (Caporaso et al., 2010) based on the 16S rRNA gene profiling analysis pipeline recommended by Pylro et al. (2014) with modifications. Forward reads were used as inputs for analysis from which barcodes were extracted followed by de-multiplexing with a Q20 filter quality (Caporaso et al., 2011). De-multiplexed sequences were further quality filtered with USEARCH 8.1 (Edgar, 2010). All sequences were truncated to a length of 143 bp. USEARCH 8.1 was further used to de-replicate sequences, sort them by their abundances and remove singletons. OTU clustering was done using the UPARSE-OTU algorithm (Edgar, 2013) to create an OTU database. Chimeric sequences were automatically discarded by the UPARSE-OTU algorithm during this step. The truncated, non-dereplicated reads were mapped back to the OTU database to create an OTU table. OTUs were classified for their taxonomy based on a 97% identity threshold using UCLUST (Edgar, 2010) and SILVA database as reference. The final OTU table was used for taxonomic annotations in the downstream analysis of community composition. Bacteria sequence reads from archaea OTU table and Archaea sequence reads from Bacteria OTU table were removed after which absolute numbers of the remaining reads in the respective OTU tables were processed for microbial community analysis (Supplementary Tables S3, S4). For graphical representation, abundance data from the OTU table was scaled to the sum of observation in each sample. The raw sequence data for this study have been submitted to GenBank Short Reads Archive (SRA) under the accession number SRP123441.

### Statistical Analysis

Significant influence of hematite and magnetite amendment to methane formation rates was inferred by performing *post hoc* analysis with Tukey procedures for each temperature and time point. The general linear hypothesis test for triplicates with default adjustments for multiple testing was applied (Hothorn et al., 2008) within the R environment (R Core Team, 2018; R version 3.4.4).

## Results

### Microbial Iron Reduction and Methanogenesis

**FIGURE 1:**
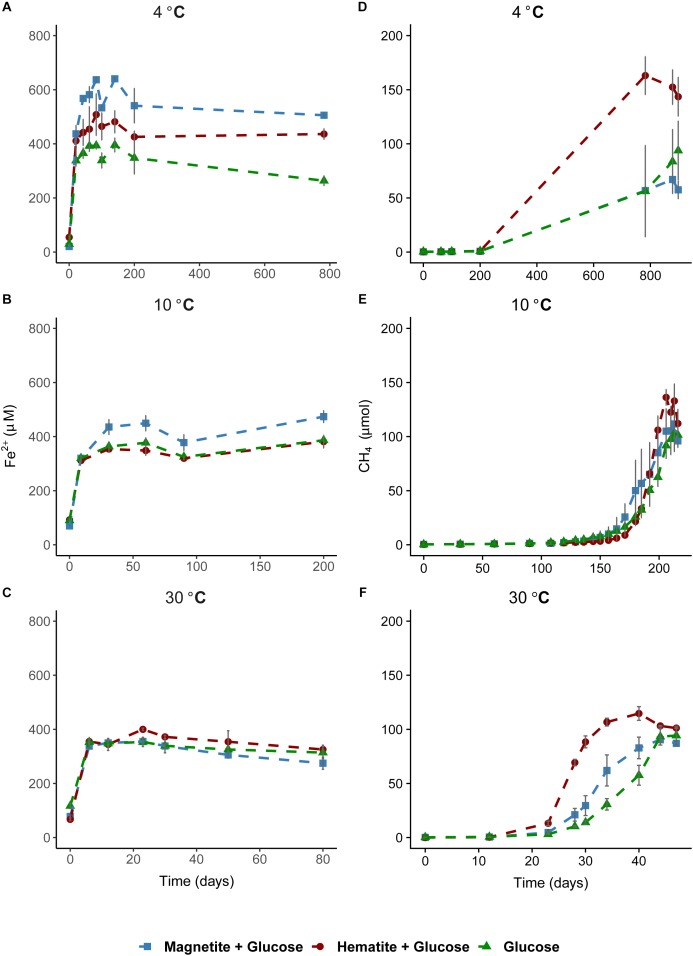
Time course of iron reduction and methane formation in sediment incubations. Left column plots show time course of iron reduction at 4°C **(A)**, 10°C **(B)**, and 30°C **(C)**. Fe^2+^ was measured over time until a stable concentration was observed in the aqueous phase across all temperatures. Right column plots show time course of methane formation at 4°C **(D)**, 10°C **(E)**, and 30°C **(F)**. In **(A)**, the 800-day time point was added to demonstrate that iron reduction was not on-going in the methanogenesis phase.

**FIGURE 2:**
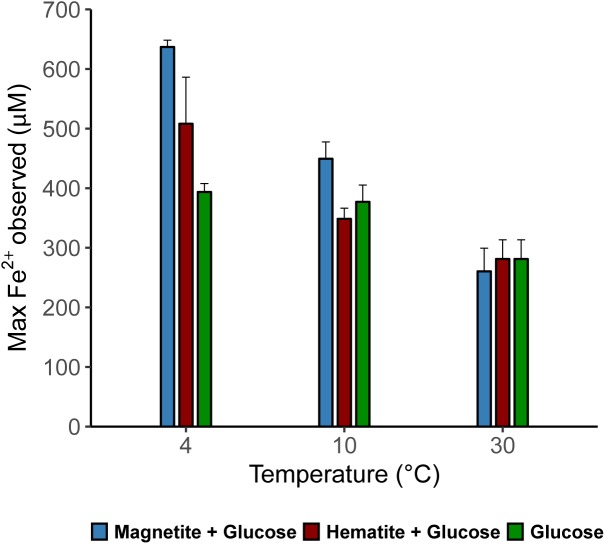
Maximum dissolved Fe (μM) measured in the various treatments across all temperatures showing the effect of crystalline iron minerals on iron reduction in sediment incubations.

Microbial iron reduction, tracked by increasing Fe^2+^ concentrations in the aqueous phase over incubation time, was evident across all temperatures (Figures 1A–C). At 4°C, Fe^2+^ concentrations were considerably higher in magnetite-glucose amended (MG) and hematite-glucose amended (HG) incubations in comparison to the “glucose only” control (G). This implies that reduction of magnetite and hematite occurred, albeit more pronounced in the case of magnetite (Figure 1A). At 10°C, iron reduction was more pronounced in MG incubations than in G and HG incubations (Figure 1B). Reduction of the crystalline iron oxides was not observed at 30°C as similar concentrations of Fe^2+^ were detected in G, MG and HG incubations (Figure 1C). In general, the potential for microbial iron reduction increased with decrease in temperature from 30 to 4°C (Figure 2). By comparing the maximum amounts of Fe^2+^ observed in each treatment (Figure 2), we could show that in the MG treatments at 4°C, 2.5-fold more Fe^2+^ were detected than at 30°C. Similarly, hematite also stimulated 1.8-fold more Fe^2+^ at 4°C compared to the same treatments at 30°C. Although additional iron oxides were not added to the G treatments, 1.4-fold more Fe^2+^ was measured at 4°C compared to 30°C.

**FIGURE 3:**
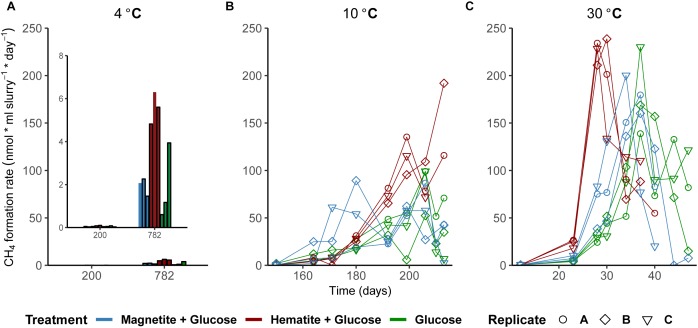
Effect of crystalline iron minerals on methane formation in sediment incubations. Methane formation rates at 4°C **(A)**, 10°C **(B)**, and 30°C **(C)**. Bar plots were displayed for methane formation rates at 4°C because fewer time-points were measured (Figure 1D).

As a measure for the occurrence of mDIET as implied in previous studies (see Lovley, 2017 and references therein), we followed methane formation in all incubations assuming that the presence of (semi)conductive iron minerals such as hematite and magnetite would facilitate more efficient electron transfer to methanogenic archaea. Methanogenesis occurred after Fe^2+^ concentrations in the aqueous phase leveled off across all temperatures (Figures 1D–F). Based on the amounts of CH_4_ measured in the headspace across all incubations and the expected stoichiometry from glucose fermentation assuming all 68 μmoles electrons from glucose is turned over to CH_4_, methanogenesis was the dominant sink for electrons across all temperatures (Table 1). At 30°C, onset of methanogenesis was observed after 23 days and continued until 47 days (Figure 1F). Methanogenesis started much later at 10°C (after 120 days) and continued until no further increase in CH_4_ amounts was detected after 216 days (Figure 1E). At 4°C, onset of methanogenesis was not observed after the initial 200 days. However, we observed after 2 years (∼780 days, Figure 1D) that methanogenesis was completed in HG incubations but continued in the MG and G incubations (898 days). Methanogenesis rates were lower with decrease in temperature from 30 to 4°C (Figures 3A–C). Across all temperatures, hematite enhanced methanogenesis in HG incubations such that both the onset and the maximum rates of methane formation were reached faster compared to G incubations (Figures 3A–C). Magnetite, similarly to hematite also enhanced the onset and maximum rates of methanogenesis; however, this was only observed at 30°C (Figure 3C). At 10°C, the enhanced onset of methanogenesis with magnetite was observed in only two of three replicates (Figure 3B). At 4°C, addition of magnetite did not enhance methanogenesis (Figure 3A). In summary, we found that hematite enhanced methanogenesis both in terms of acceleration and maximum process rates, whereas the magnetite only improved its acceleration (i.e., the time between process onset and reaching maximum process rates), compared to incubations without iron oxide amendments.

**Table 1:** Average amounts of methane formed and maximum methanogenesis rates per treatment across temperatures.

Temperature	Treatment	Average maximum CH_4_ amount measured (μmol)	% Expected CH_4_ accounted for	Maximum CH_4_ formation rate (nmol ^∗^ ml slurry^-1^ day^-1^)
4°C	Glucose	93.8 ± 27.5	46	1.9 ± 1.5
	Magnetite + Glucose	67 ± 5.8	32.9	1.9 ± 0.3
	Hematite + Glucose	163.1 ± 17.8	80	5.6 ± 0.6
10°C	Glucose	103.2 ± 17	50.6	83.5 ± 22
	Magnetite + Glucose	111.7 ± 16.3	54.8	79 ± 12.7
	Hematite + Glucose	136.3 ± 7.2	66.8	141.1 ± 36
30°C	Glucose	94.3 ± 8.8	46.2	179.2 ± 38
	Magnetite + Glucose	90.4 ± 2.2	44.3	180 ± 12.7
	Hematite + Glucose	114.7 ± 6.3	56.2	233.7 ± 4.2


In order to assess the role of methanogenesis in electron flow through iron oxides, methanogenesis was specifically inhibited by adding BES (15 mM) to incubations (30°C). Based on the Fe^2+^ concentrations measured, there was no difference in the rates of iron reduction in the presence of hematite and magnetite when compared to the glucose + BES incubations (Supplementary Figure S2).

### Microbial Community Shifts at Varying Incubation Temperatures

**FIGURE 4:**
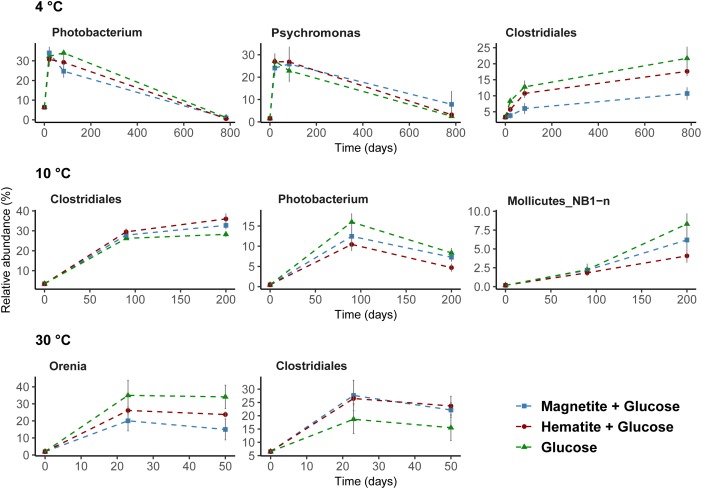
Dominant bacteria communities enriched over time at 4°C, 10°C, and 30°C determined by 16S rRNA gene analysis. A threshold of relative abundance increase of 5% was used to determine key taxa (from order to genus level) compared to controls. Percentages show relative abundance of individual genus or order. Data presented here are reflecting the main bacteria taxa that were stimulated by glucose addition to slurry incubations.

Microbial community composition in slurry incubations was analyzed in order to identify key microorganisms involved in glucose and iron oxide utilization. Because of the different temperature regimes applied, microbial activities were different, which is reflected in the different time intervals of sampling (see Supplementary Figures S3–S5). At 4°C, the genera *Photobacterium* and *Psychromonas* were dominant and their relative abundance was highest after 21 days (ranging from 24 to 34%, respectively; Figure 4). While sequences related to *Psychromonas* were not observed at 10°C, *Photobacterium* spp. were enriched (10–15% after 90 days; Figure 4). Both *Psychromonas* and *Photobacterium* populations were absent at 30°C; Figure 4. Members of the order Clostridiales were observed across all temperatures and were mostly dominant at 10°C (up to 36% at day 90). Within the order Clostridiales, the genus *Fusibacter* was observed across all temperatures (Supplementary Figure S7). However, there were differences in the other dominant Clostridiales family or genus observed at the different temperatures. For example, *Alkalibacter* spp. was low in relative abundance at 4°C (<1%); enriched at 10°C (3–6% after 90 days) in all treatments and was only observed in the magnetite amended treatment at 30°C (4%, day 23). Family JTB 215 and Clostridiaceae_1 were only enriched at 30°C (Supplementary Figure S7). GoM-GC232-4463-Bac1 was enriched at 10°C and 30°C but not at 4°C (Supplementary Figure S7). Representatives of the order NB1-n (phylum Tenericutes) were present, but only at 10°C after 200 days (4–8%; Figure 4). At 30°C, members of the genus *Orenia* were enriched (26–36% after 23 days; Figure 4) but were not observed under psychrophilic conditions.

**FIGURE 5:**
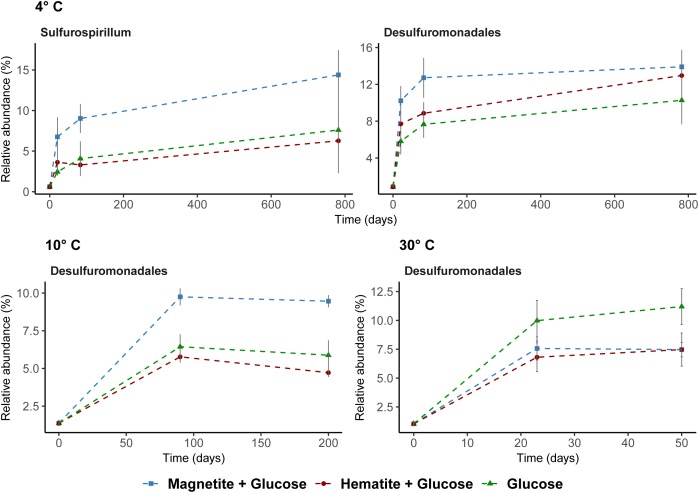
Potential iron reducing bacteria based on 16S rRNA gene analysis across all temperatures. *Sulfurospirillum* shown at 4°C only; were below 1% relative abundance at other temperatures.

Known iron-reducing taxa detected in enrichments were related to the order Desulfuromonadales and the genus *Sulfurospirillum* (family Campylobacteraceae, class Epsilonproteobacteria) (Figure 5). At low temperatures, where we obtained indications that magnetite (4°C and 10°C) and hematite (4°C) reduction occurred, members of the order Desulfuromonadales were more abundant in MG and HG compared to the control, G (Figure 4). While Desulfuromonadales were also present at 30°C, their relative abundance was lower in the iron oxide amendments (Figure 5). This correlated with the observations that hematite and magnetite were not reduced at 30°C (Figure 1). Within Desulfuromonadales, genus *Desulfuromonas* was the dominant taxa enriched at 4°C and 10°C (up to 11%; Supplementary Figure S8). In contrast at 30°C, the genus *Pelobacter* was dominant (up to 10%; Supplementary Figure S8). The relative abundance of *Pelobacter* was less than 0.5% at 4°C and 10°C (Supplementary Figure S8). *Desulfuromonas* were only enriched in the magnetite amended treatment (4.2 ± 0.5% at day 50) at 30°C. At 4°C, the relative abundance of members of the genus *Sulfurospirillum* in MG was higher after 83 days (9 ± 1.8%) compared to HG (3 ± 0.2%) and G (4.08 ± 2%). *Sulfurospirillum* was not enriched at 10°C or 30°C.

**FIGURE 6:**
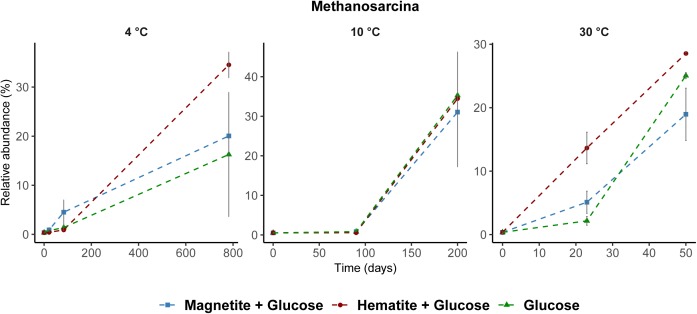
Relative abundance of *Methanosarcina* spp. across all temperatures determined by archaea 16S rRNA gene analysis. *Methanosarcina* was the only known methanogen that increased in relative abundance during methane formation regardless of incubation temperature.

Among Archaea, *Methanosarcina* were the only methanogens enriched across all incubations regardless of incubation temperature (Figure 6). Generally, the relative abundance of other Archaea taxa did not change except at time-points when methanogenesis occurred and *Methanosarcina* was enriched (Supplementary Figures S3–S5).

## Discussion

### Crystalline Iron Oxides Utilization Under Temperature Control

Iron oxide minerals are known as electron acceptors but their important role as conductors for facilitating microbial interspecies electron transfer was only recently recognized (Kato et al., 2012; Jiang et al., 2013; Cruz Viggi et al., 2014; Zhou et al., 2014). Conversely, factors are unknown that determine whether iron oxides are reduced in dissimilatory fashion or used to facilitate electron flow between microbial populations in the environment. Here, we demonstrate that temperature has a pronounced effect on crystalline iron oxide utilization in marine sediment incubations. A shift from low (i.e., 4–10°C) to high temperature (i.e., 30°C) resulted in lower iron reduction rates indicated by higher amounts of Fe^2+^ detected at lower temperatures (Figure 2). Consequently, electron transfer toward methanogenesis was enhanced in the presence of hematite or magnetite, especially at 30°C (Figures 3A–C). Thus, temperature appears to be a prime regulator controlling the mode (reduction or conduction) of iron oxide utilization by microorganisms in marine sediments. The underlying mechanisms are most likely multifactorial encompassing (1) community composition changes, (2) the reaction thermodynamics and pathways of degradation involved, and (3) specific adaptations to temperature.

Firstly, temperature is known to have a strong effect on metabolic adaptation and microbial community composition, both, for planktonic and benthic microorganisms (Fuhrman, 2009; Robador et al., 2009; Blake et al., 2015). Bottom water temperatures overlying the sediments of the Helgoland Mud Area are fluctuating annually between 2°C in March and 19°C in August (Oehler et al., 2015). Thus, incubations at 4°C and 10°C reflected seasonal *in situ* temperatures, whereas 30°C reflected a strong temperature shift. Not surprisingly, induced by different temperature regimes and glucose amendment, different key microorganisms were enriched (Supplementary Figures S3–S5) similar to other studies (Upton et al., 1990; Robador et al., 2009; Adams et al., 2010; Blake et al., 2015). In addition to their largely different taxonomic affiliations, temperature selected for communities that differed in their capabilities for crystalline iron oxide utilization (Figures 2, 3). Secondly, thermodynamics of the reactions involved seems to favor different physiological guilds competing for common electron donors. For example, methanogenesis is known to be thermodynamically more feasible under warmer (mesophilic) temperatures (Zeikus and Winfrey, 1976; Van Hulzen et al., 1999; Fey and Conrad, 2000; Yao and Conrad, 2000) than under lower temperatures (Conrad and Wetter, 1990), and can have a strong effect on carbon fractionation in temperature dependent methanogenesis (Fey et al., 2004). Nevertheless, methanogenic activity in freshwater and arctic wetland sediments has been observed over a wide temperature range from 1 to 45°C (Zeikus and Winfrey, 1976; Franzmann et al., 1992; Simankova et al., 2003; Blake et al., 2015). Some methanogenic isolates are psychrophiles. For example, *Methanogenium frigidum*, isolated from Ace Lake in Antarctica, which grows optimally at 15°C but not above 18–20°C (Franzmann et al., 1997). Sequences of methanogenic archaea were detected in sediments of the Helgoland Mud Area (Oni et al., 2015b) suggesting a potential for methanogenesis in these sediments. Although we did not observe methanogenesis at 4°C during the initial time-course of the experiments (200 days), its subsequent occurrence was only a matter of incubation time (∼780 days). *Methanosarcina* were the only methanogens enriched from our incubations regardless of incubation temperature or addition of crystalline iron oxides (Figure 6) and majority of the electrons from glucose fermentation ended up being used by *Methanosarcina* for methane formation (Table 1). Microorganisms are known for specific temperature adaptations such as membrane fluidity, composition and expressed amount of enzymes, as well as specific regulation response for coping with temperature stress (D’Amico et al., 2006; De Maayer et al., 2014). Our results show that microbial iron reduction was more feasible at lower temperatures (4°C and 10°C) than at 30°C, to the extent that both amended crystalline iron minerals were reduced at 4°C (Figures 1A, 2). When methanogenesis was BES inhibited in 30°C incubations (Supplementary Figure S2), reduction of magnetite and hematite still remained low. Similar Bacteria communities were observed in the BES incubations when compared to the 30°C incubations without BES (Supplementary Figures S5, S6). Thus, the absence of enhanced iron reduction with magnetite or hematite addition at 30°C was not due to the onset and transfer of electrons from glucose fermentation to methanogenesis. Rather, the microorganisms enriched in the sediments at 4°C and 10°C are better adapted to perform crystalline iron oxide reduction than those enriched at 30°C. Similarly, iron reduction rates increased with a decrease in temperature from 15 to 4°C in ferrihydrite reducing slurry incubations from glacial sediments (Nixon et al., 2017).

The observed strong increase in methanogenesis rates (up to twofold) in the presence of hematite (Figures 1, 3) suggests that (semi)conductive hematite may have served as conduit in our marine sediment incubations, facilitating mDIET. This is also the first study that demonstrates that mineral mediated enhancement of methanogenesis can occur at psychrophilic temperatures down to 4°C. Therefore, mDIET based enhancement of methanogenesis, which has only been previously demonstrated in enrichments from rice field soils and river sediments (see Lovley, 2017); can also enhance methanogenesis in cold marine sediments. Electrons from glucose fermentation were likely shunted to methanogens via the (semi)conductive, crystalline iron minerals (i.e., hematite and magnetite). Similarly, Kato et al. (2012) found an enhancement of methane formation in rice field soil incubations, which was accompanied by stimulated growth of *Geobacter* spp. in incubations amended with hematite and magnetite. Comparable effects on methanogenesis were observed with magnetite at 30°C, but at 10°C and 4°C, magnetite served preferentially as an electron acceptor than as a conduit (Figures 1–3). At 4°C, hematite apparently played a dual role, acting initially, as an electron acceptor and subsequently facilitating electron transfer when the conditions were favorable for methanogenesis to occur. More Fe^2+^ was observed in hematite + glucose incubations compared to the glucose amended control but methanogenesis was subsequently enhanced in hematite amended incubations (Figures 1A,D, 3A). Likewise, Zhou et al. (2014), in rice field soil enrichments incubated at 30°C, observed a similar effect in the presence of magnetite.

Since sediment incubations are less defined than pure culture studies, other potential mechanisms could have been operative in addition to or instead of mDIET. Magnetite could have served as electron acceptor (Yang et al., 2015), or as part of an iron redox cycle, could have been important in the production of H_2_ from acetate to support hydrogenotrophic methanogenesis (Jiang et al., 2013); however, iron reduction was not observed in our incubations concurrently while methanogenesis was on-going (Figure 1). More research is required to elucidate the exact mechanism for enhanced methanogenesis in the presence of crystalline iron oxides.

### Crystalline Iron Oxide Reducing Bacteria Under Psychrophilic Conditions

Due to easier accessibility by microorganisms and thermodynamic favorability (Weber et al., 2006), poorly crystalline iron minerals have been mostly used in studying iron reduction in samples from sedimentary environments (Roden and Lovley, 1993; Zhang et al., 1999; Vandieken et al., 2006; Hori et al., 2015). This has led to the lack of knowledge of the diversity of microorganisms capable of reducing crystalline iron minerals, despite their abundance in natural environments (Hori et al., 2015).

The increase in the relative abundance of *Sulfurospirillum* with increasing concentration of Fe^2+^ (Figures 1A, 5) implies their involvement in dissimilatory reduction of magnetite at 4°C. Members of the genus *Sulfurospirillum* have been previously linked to growth with poorly crystalline iron oxides at mesophilic temperatures. For example, *Sulfurospirillum barnesii* can use amorphous Fe(III) and ferrihydrite as terminal electron acceptors (Stolz et al., 1999; Zobrist et al., 2000) while *S. deleyianum* is capable of ferrihydrite dependent growth coupled to sulfur cycling (Straub and Schink, 2004; Lohmayer et al., 2014). The increase in relative abundance of Desulfuromonadales over time in the 4°C incubations correlated with the iron reduction kinetics, i.e., a higher relative abundance of Desulfuromonadales sequences was observed in incubations with higher Fe^2+^ concentration (Figures 1A, 5). At 10°C in incubations with added magnetite, a higher abundance of Desulfuromonadales sequences also correlated with the higher Fe^2+^ concentrations measured (Figures 4, 1B). Along with the different temperature regimes, the dominant genus enriched within the Desulfuromonadales order were clearly different. *Desulfuromonas* was dominant at lower temperatures while *Pelobacter* was dominant at 30°C (Supplementary Figure S8). *Desulfuromonas* species are capable of reducing poorly crystalline iron oxides in marine surface sediments (Roden and Lovley, 1993; Vandieken et al., 2006). Here, we show they are involved in magnetite reduction as well under psychrophilic conditions in the marine sub-surface.

### mDIET-Linked Microorganisms Under Mesophilic and Psychrophilic Conditions

Most known members of the family Halobacteroidaceae ferment carbohydrates to acetate, ethanol, H_2_, and CO_2_ (Oren, 2014). Besides, some species are homoacetogenic but can also use a variety of electron acceptors, e.g., selenate, arsenate, and iron oxides (Oren, 2014). Therefore, the dominance of the genus *Orenia*, regardless of the presence or absence of the crystalline iron oxides within the enriched communities at 30°C (Figure 4), suggests that members of the genus *Orenia* were largely responsible for glucose fermentation. In addition, *Orenia* might have been involved in iron reduction together with *Pelobacter* spp., who are capable of fermentative and dissimilatory Fe(III) reduction (Lovley et al., 1995). Syntrophic interactions that occur between microbes and iron minerals during methanogenic fermentation of organic matter mediate the electron transfer during these interactions (Kato et al., 2012; Yang et al., 2015). Although an isolated species within the genus, *Orenia metallireducens* strain Z6, can reduce both poorly crystalline and crystalline iron oxides (Dong et al., 2016), the reduction of amended magnetite and hematite was not detectable based on a lack of increasing Fe^2+^ concentrations at 30°C. Thus, it is likely that the enriched members of the genus *Orenia* detected here shuttled electrons from glucose fermentation to *Methanosarcina* via mDIET to the amended hematite or magnetite; this in turn accelerated the onset of methane formation and enhanced the process rates (Figure 3C).

The enrichment of members of the known psychrophilic genera *Photobacterium* and *Psychromonas* at 4°C might be linked to glucose fermentation (Seo et al., 2005; Auman et al., 2006). Enrichment of different Clostridiales sub-groups at different temperatures (Supplementary Figure S7) demonstrates the versatility of the order Clostridiales to thrive at various temperature regimes. The order Clostridiales is well known for harboring a wide variety of fermenting microorganisms. For example, the genus *Fusibacter* which was enriched across all temperatures studied (Supplementary Figure S7) has been shown to be capable of glucose metabolism (Ravot et al., 1999; Hania et al., 2012; Fadhlaoui et al., 2015; Smii et al., 2015). Some organisms within the order Clostridiales are also exoelectrogens (Jiang et al., 2013; Fuller et al., 2014; Moscoviz et al., 2017), thus, they can potentially transfer electrons to methanogens directly via mDIET in our incubations. This in turn, may have resulted in the enhanced rates of methanogenesis observed at 10°C and 4°C.

## Conclusion

Our results open a new window into understanding environmental regulators of microbial interaction and utilization of crystalline iron minerals. We identified temperature as one of the regulators important for the mode of crystalline iron mineral utilization by microorganisms. We also demonstrate the potential for mDIET to occur in sub-surface marine sediments and at temperatures down to 4°C. Thus, crystalline iron oxides may facilitate electron transfer between microorganisms thriving in anoxic cold sediments in addition to serving as electron acceptors. This extended role of crystalline iron oxides in microbial metabolism could have an impact on the biogeochemical cycling of carbon in sedimentary environments by accelerating the rate of organic carbon biomineralization. More work is certainly required to identify specific molecular adaptations to iron reduction or utilization as conduit under temperature control.

## Author Contributions

DA, OO, TR-H, and MF designed the experiments. DA performed the experiments and data analysis with support from OO, AK, XY, TR-H, and MF. MF secured funding for this research. SK carried out the HE433 expedition and provided sediment samples for the experiments. DA (90%) and MF (10%) wrote the manuscript with contributions from all co-authors.

## Conflict of Interest Statement

The authors declare that the research was conducted in the absence of any commercial or financial relationships that could be construed as a potential conflict of interest.
