# Supplementary material for: Temperature Controls Crystalline Iron Oxide Utilization by Microbial Communities in Methanic Ferruginous Marine Sediment Incubations
Source: Front Microbiol. 2018 Oct 30;9:2574. doi: 10.3389/fmicb.2018.02574 (PMC6218420; doi:10.3389/fmicb.2018.02574)
Supplement: Supplementary file 1 [file Data_Sheet_1.pdf]

Supplementary Information

Temperature Controls Crystalline Iron Oxide Utilization by Microbial Communities in Methanic Ferruginous Marine Sediment Incubations

David A. Aromokeye[1,2,3], Tim Richter-Heitmann[1], Oluwatobi E. Oni[1,2], Ajinkya Kulkarni[1,2,3], Xiuran Yin[1,2,3], Sabine Kasten[2,4,5] and Michael W. Friedrich[1,2,*].

[1]Microbial Ecophysiology Group, Faculty of Biology/Chemistry, University of Bremen, Bremen, Germany

[2]MARUM, Center for Marine Environmental Sciences, University of Bremen, Bremen, Germany

[3]International Max-Planck Research School for Marine Microbiology, Max-Planck-Institute for Marine Microbiology, Bremen, Germany

[4]Alfred Wegener Institute Helmholtz Centre for Polar and Marine Research, Bremerhaven, Germany

[5]University of Bremen, Faculty of Geosciences, Bremen, Germany

*Correspondence:

Michael W. Friedrich,

[1]Microbial Ecophysiology Group, Faculty of Biology/Chemistry & MARUM, University of Bremen, PO Box 33 04 40, D-28334 Bremen, Germany

Email: michael.friedrich@uni-bremen.de

Supplementary Figures

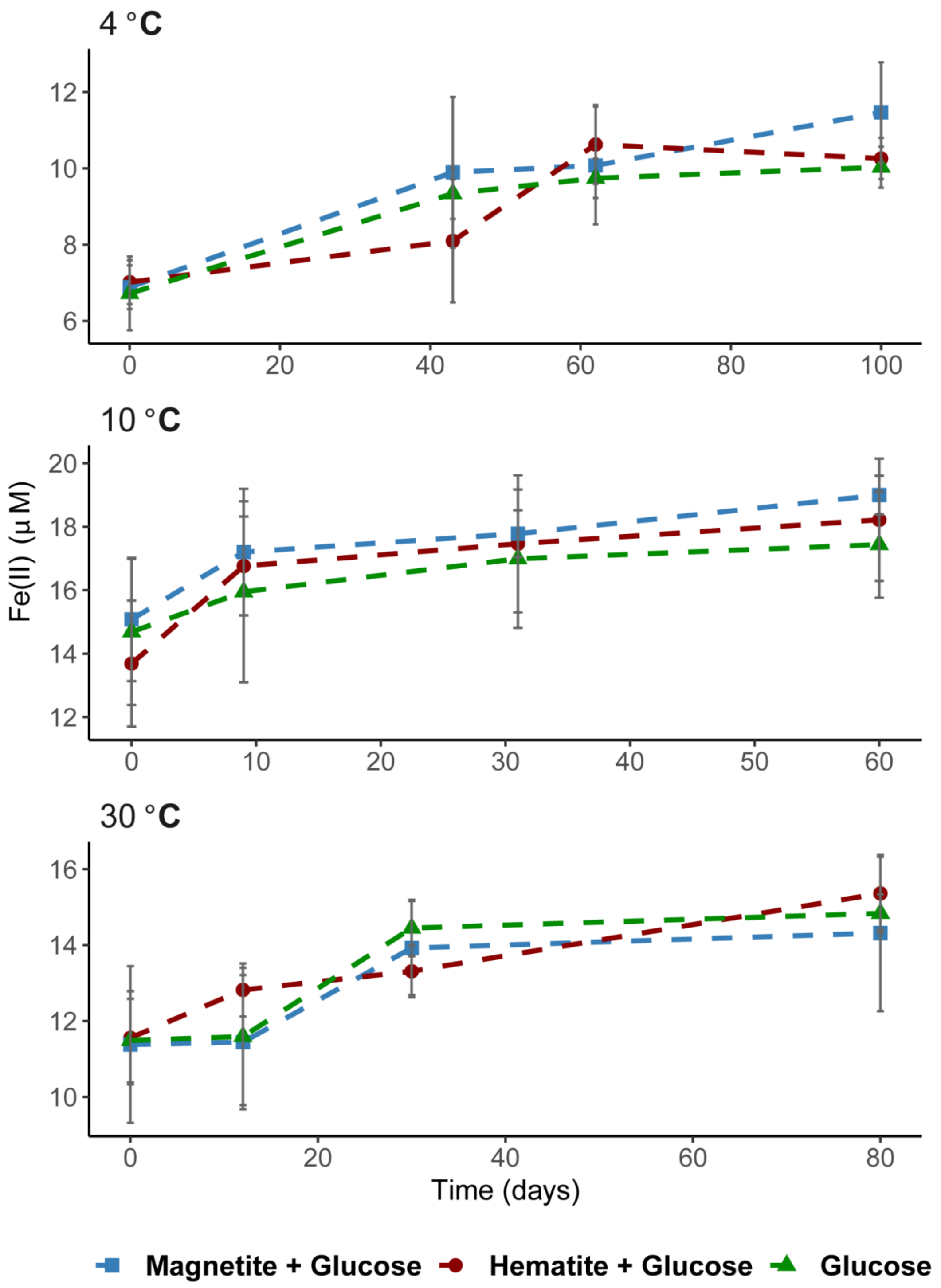

Figure S1: HCl extractable Fe(II) measured across selected time-points during the iron reduction experiments. The total Fe(II) measurements were done on whole slurry material (see methods) unlike the Fe^{2+} measurements from pore water (Fig. 1, main text).

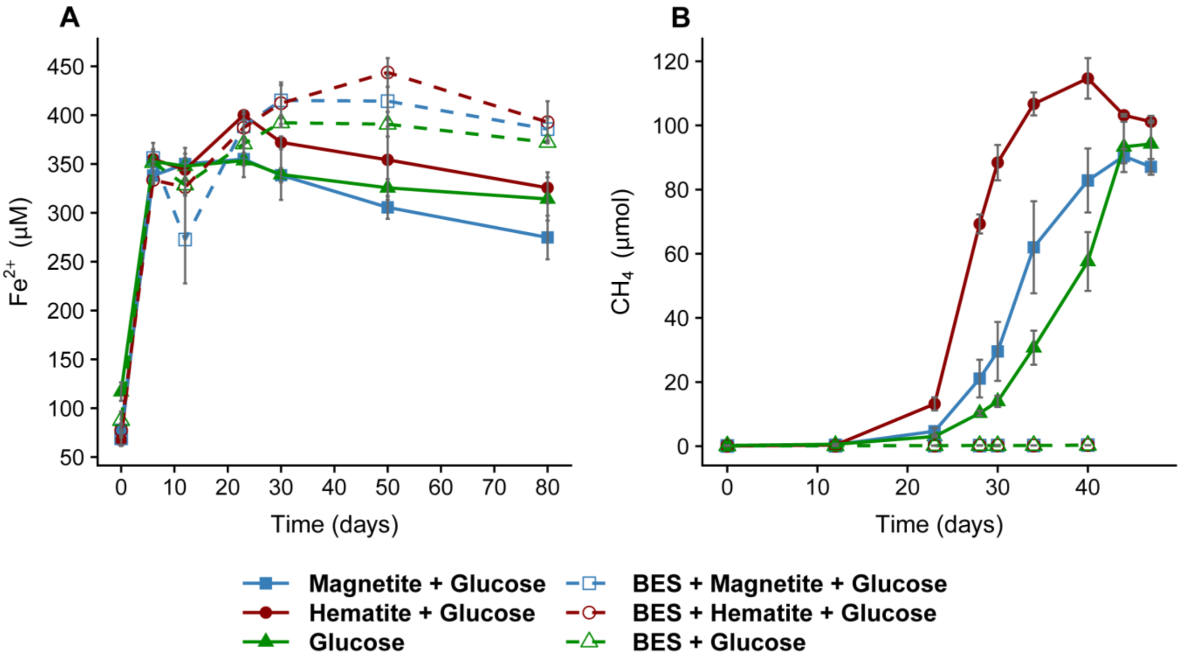

Figure S2: Effect of BES on iron reduction (A) and methanogenesis (B) in the presence of the crystalline iron minerals. Incubations were performed at 30 °C. Data presented for the non BES amended controls are presented in Figure 1 of the main text but are re-plotted here as controls for evaluating the effect of BES on iron reduction in comparison to the controls.

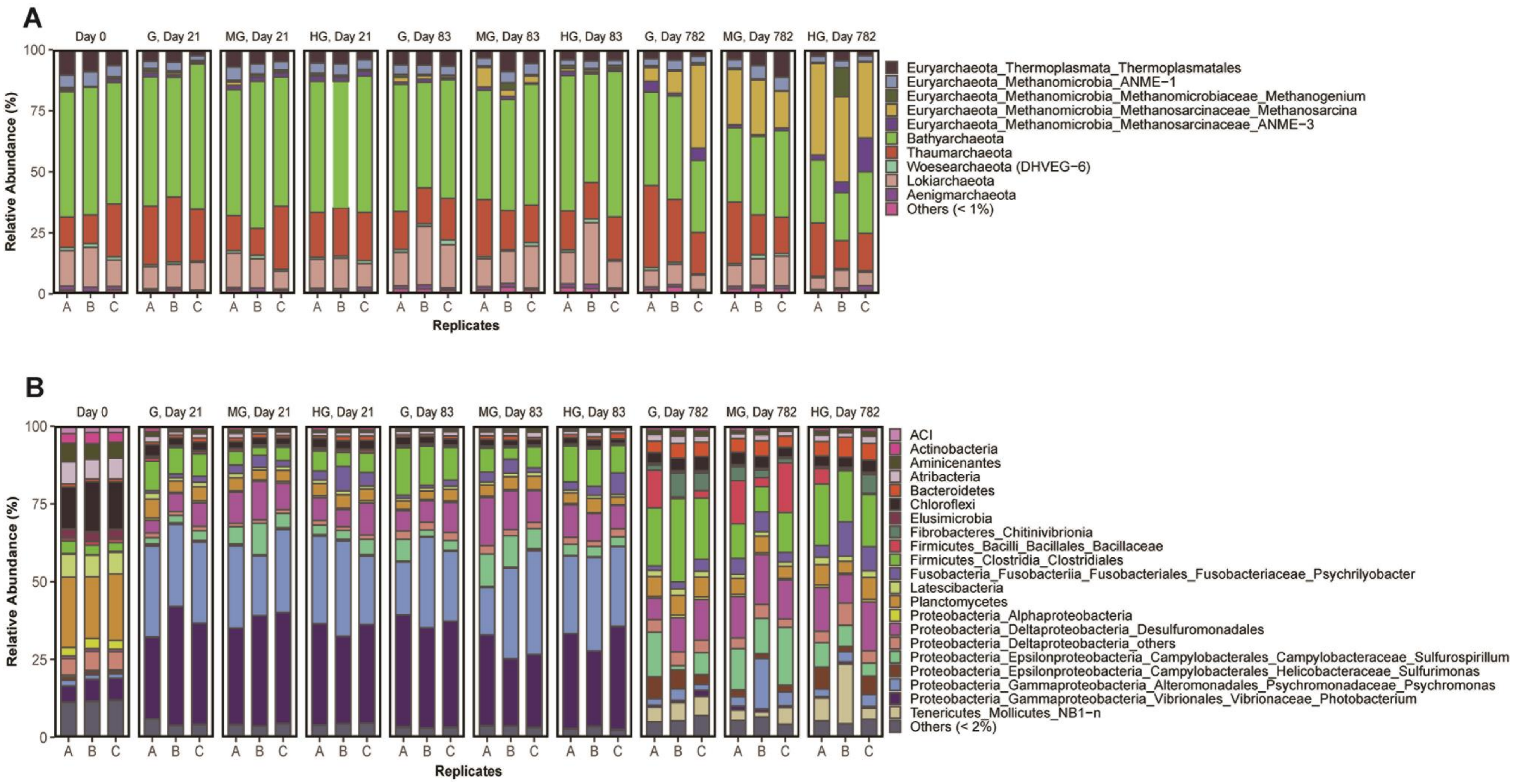

Figure S3: Total sum scaling of archaea (A) and bacteria (B) communities in incubations at 4 ºC between day 0, day 21 (intermediate iron reduction phase), day 83 (peak of iron reduction phase based on Fe^{2+} concentrations) and day 782 (methanogenesis phase). G, glucose amended incubation; MG, incubations amended with magnetite and glucose; HG, hematite and glucose amendments. Each triplicate sample was sequenced individually (see methods). Microbial communities were scaled in a mixed style such that the phyla with decreased relative abundance over time compared to day 0 were reported on the phylum level. Lower taxonomic classification was reported for orders, families and genera from other phyla with increased relative abundance compared to day 0.

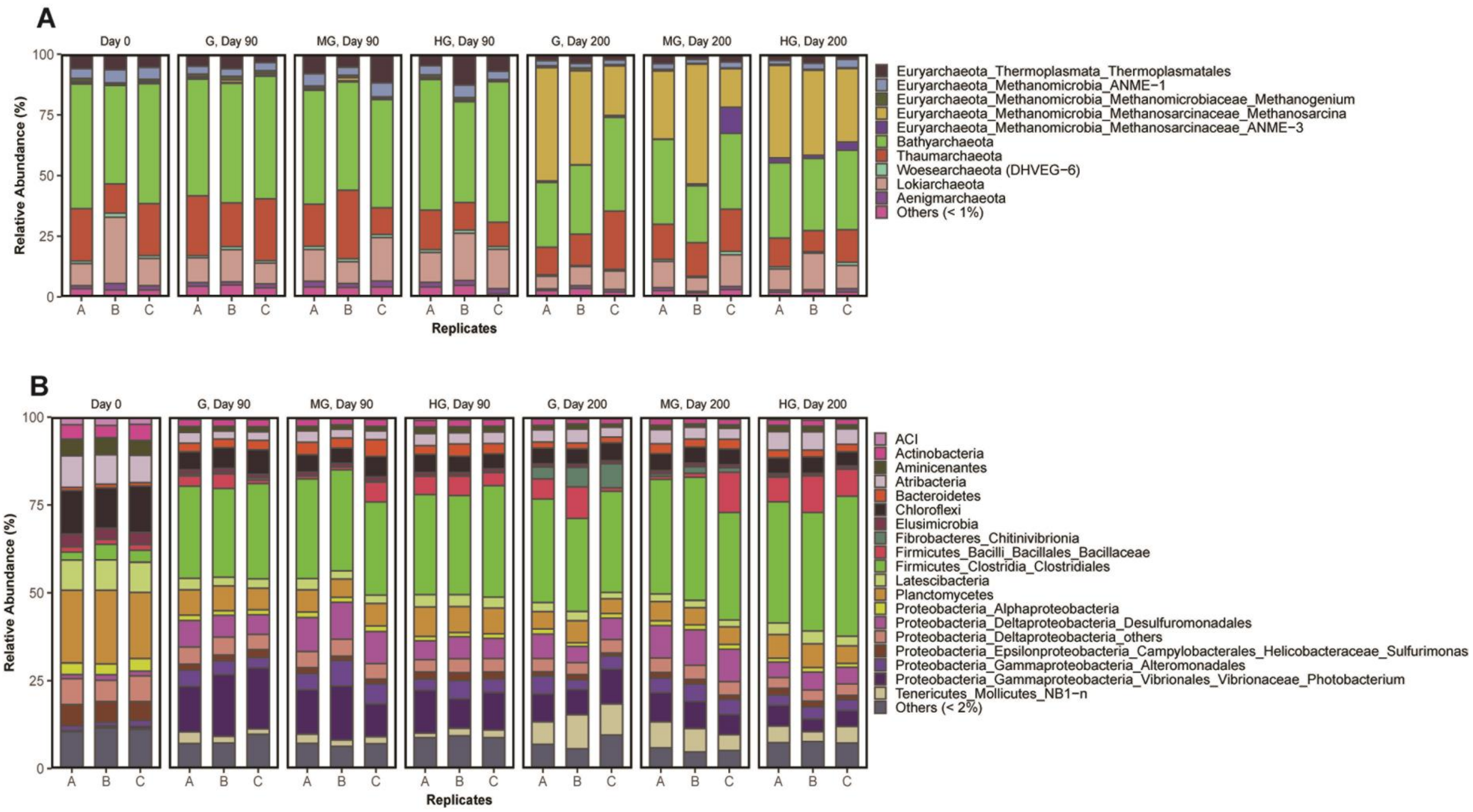

Figure S4: Total sum scaling of archaea (A) and bacteria (B) communities in the incubations at 10 ºC between day 0, day 90 (when aqueous Fe^{2+} concentrations reached a plateau) and day 200 (during active methanogenesis). G represents glucose amended incubation; MG represents incubations amended with magnetite and glucose while HG represents hematite and glucose amendments. Each triplicate sample was sequenced individually (see methods). Microbial communities were scaled in a mixed style such that the Phyla with decreased relative abundance over time compared to day 0 were reported on the phylum level. Whereas, lower taxonomic classification was reported for orders, families and genera from other phyla with increased relative abundance compared to day 0.

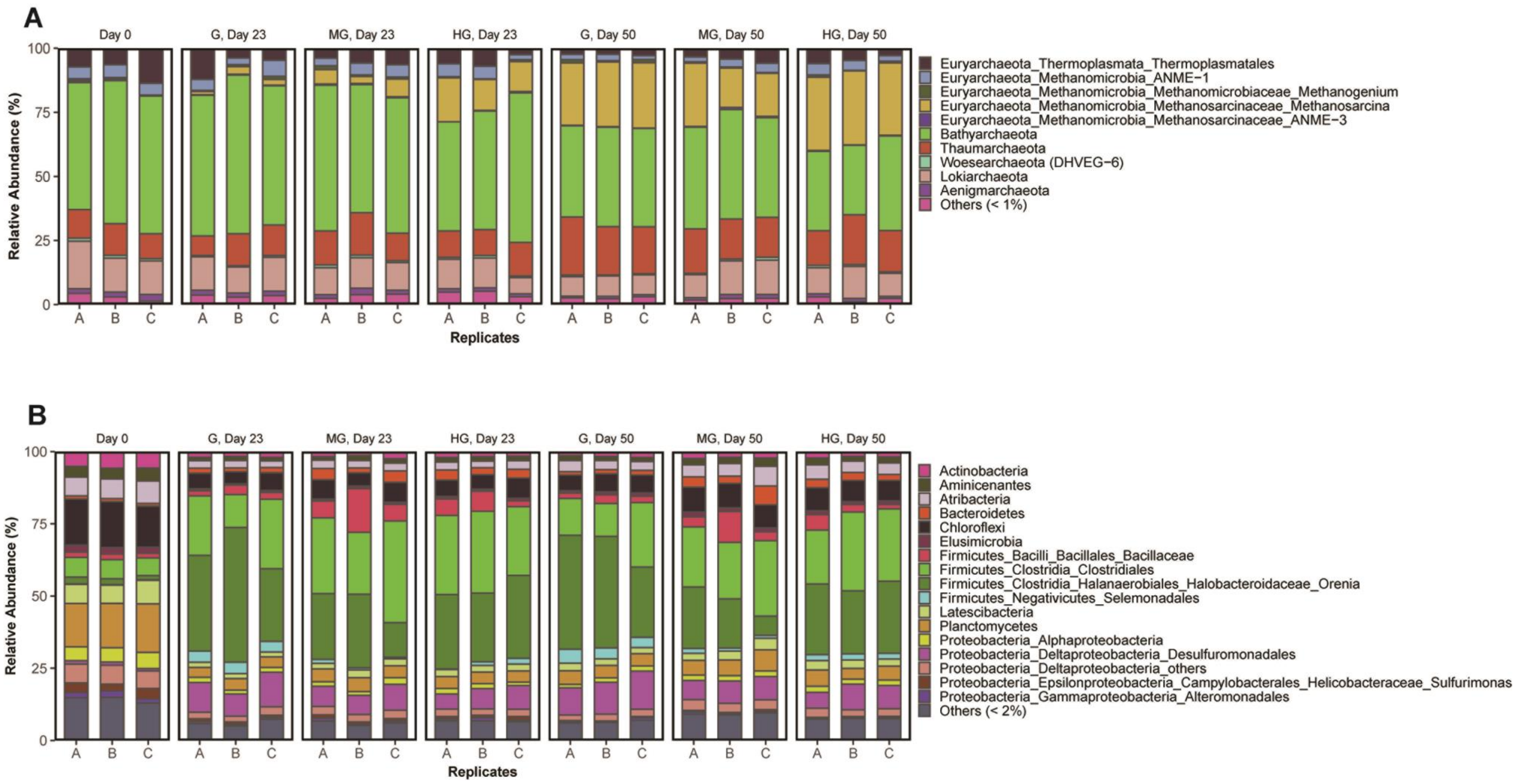

Figure S5: Total sum scaling of bacteria (A) and archaea (B) communities in the incubations at 30 ºC between day 0 day 23 when Fe^{2+} formation reached a plateau and at day 50, when methane concentrations levelled off. G represents glucose amended incubation; MG represents incubations amended with magnetite and glucose while HG represents hematite and glucose amendments. Each triplicate sample was sequenced individually (see methods). Microbial communities were scaled in a mixed style such that the Phyla with decreased relative abundance over time compared to day 0 were reported on the phylum level. Whereas, lower taxonomic classification was reported for orders, families and genera from other phyla with increased relative abundance compared to day 0.

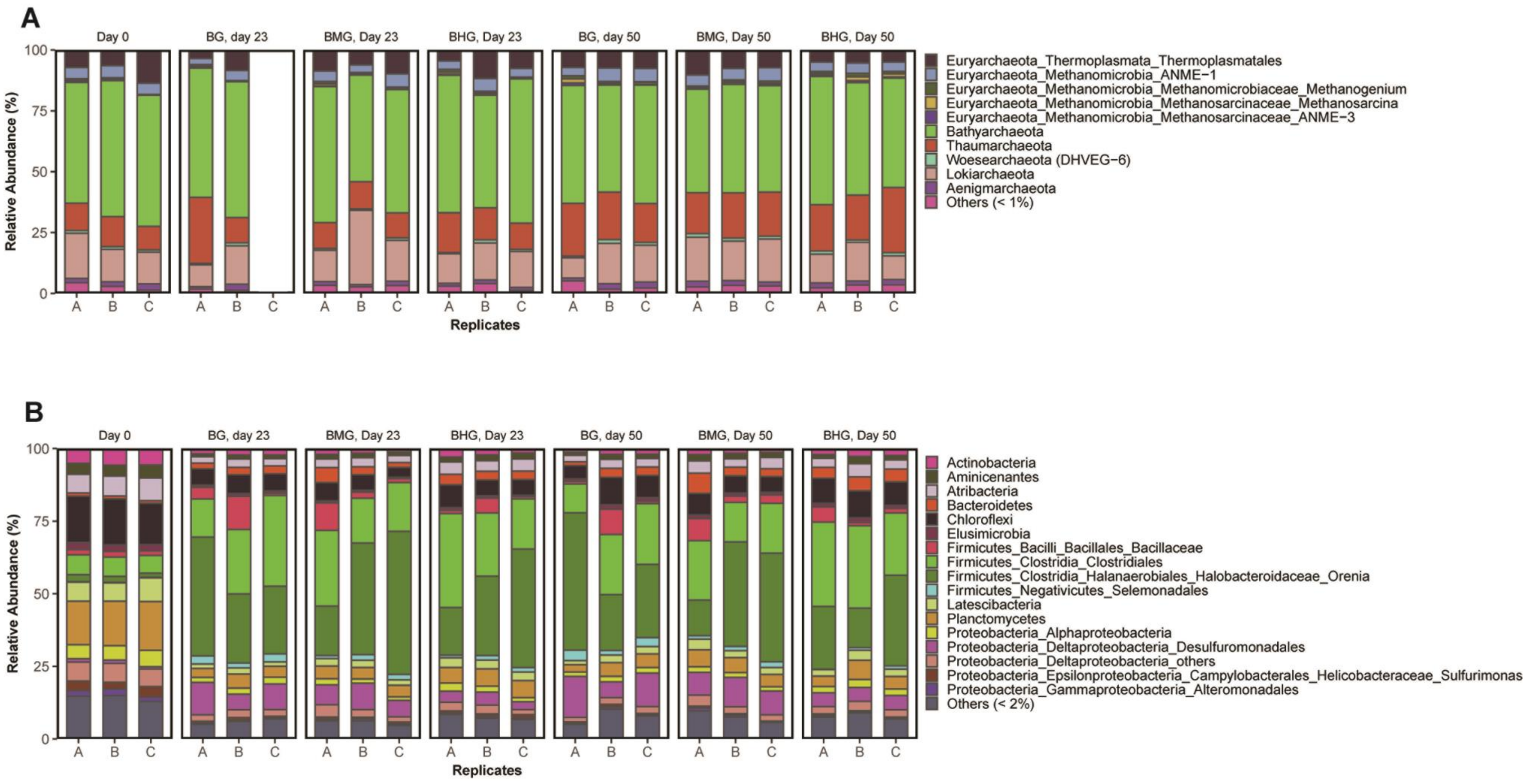

Figure S6: Total sum scaling of bacteria (A) and archaea (B) communities in the incubations amended with BES at 30 ºC. The archaea sample for BG.c at day 23 failed during the sequencing preparation, hence triplicates for that time point could not be shown.

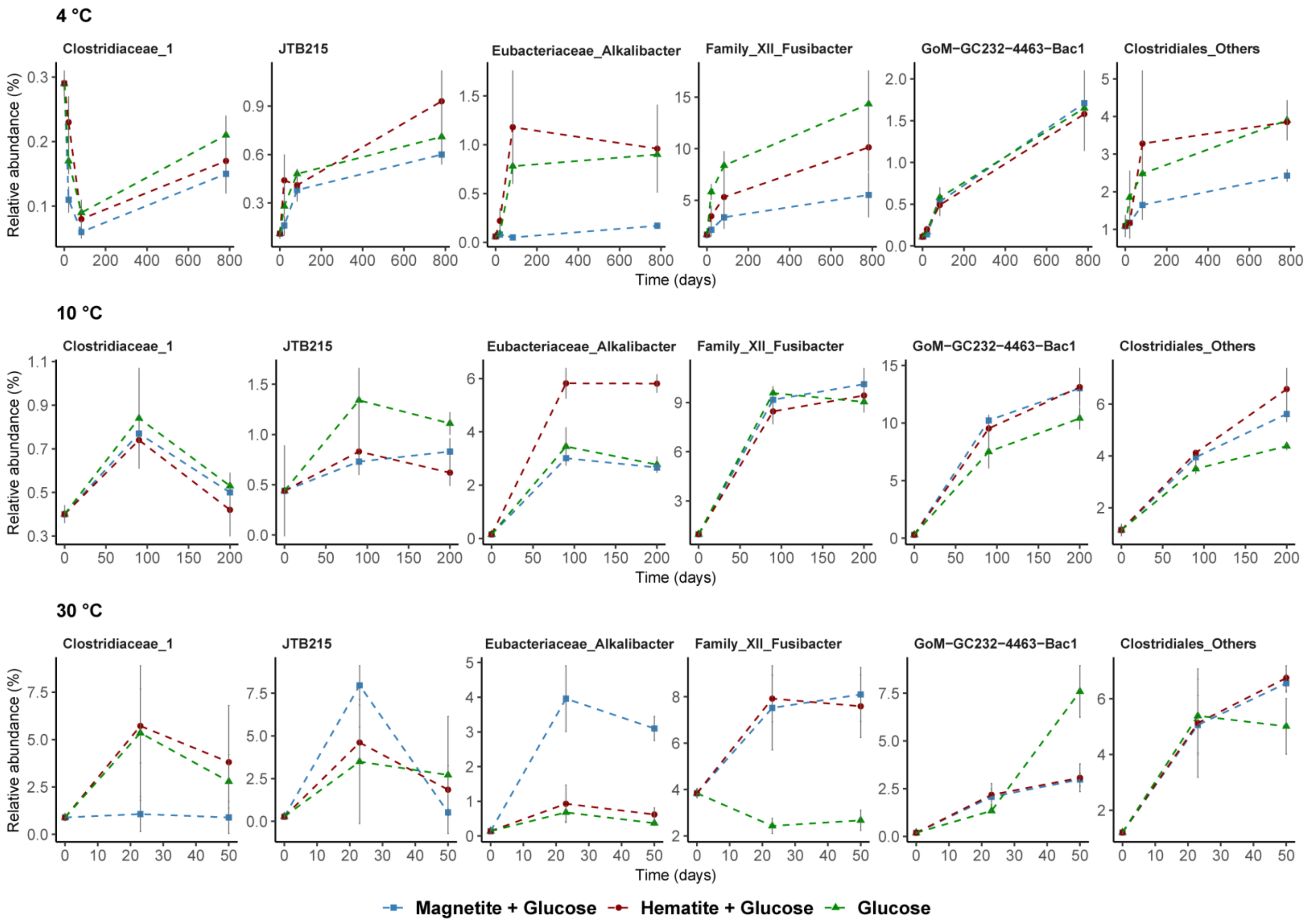

Figure S7: Relative abundance of genus or family belonging to the order Clostridiales that were identified at different temperatures demonstrating the versatility of the order Clostridiales at various temperatures**.**

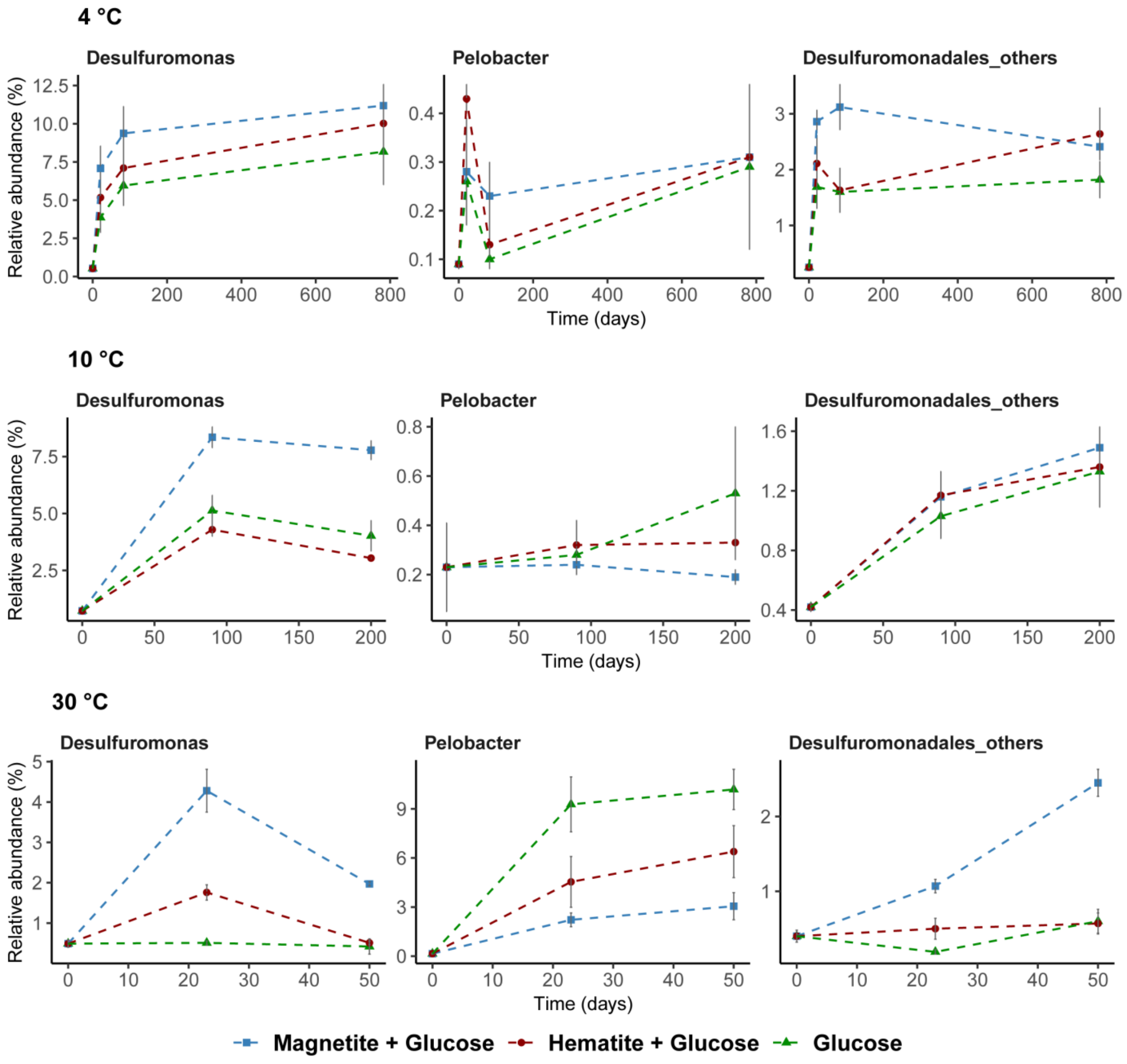

Figure S8: Relative abundance of dominant genus from the order Desulfuromonadales. *Desulfuromonas* was higher in relative abundance at 4 ºC and 10 ºC while *Pelobacter* was more enriched at 30 ºC.

Table S1. Summary of triplicate incubation experiment set up to study microbial iron reduction and methanogenesis.

Treatment (n=3)	Temperature (^{o}C)	Glucose (68 µmoles)	Magnetite (1020 µmoles)	Hematite (1020 µmoles)	Sodium 2-bromoethane sulfonate (BES, 15mM)	Sediment depth used (cm)	Incubation duration (days)
Magnetite + Glucose	4	+	+	-	-	441–466	900
Hematite + Glucose	4	+	-	+	-	441–466	900
Glucose	4	+	-	-	-	441–466	900
Magnetite + Glucose	10	+	+	-	-	441–466	216
Hematite + Glucose	10	+	-	+	-	441–466	216
Glucose	10	+	-	-	-	441–466	216
Magnetite + Glucose	30	+	+	-	-	416–441	80
Hematite + Glucose	30	+	-	+	-	416–441	80
Glucose	30	+	-	-	-	416–441	80
Magnetite + Glucose + BES	30	+	+	-	+	416–441	80
Hematite + Glucose + BES	30	+	-	+	+	416–441	80
Glucose + BES	30	+	-	-	+	416–441	80

Table S2. pH of incubations in duplicate supplementary set-ups to check the effect of glucose fermentation on the slurries.

Time (days)	Temperature	Magnetite + Glucose_ A	Magnetite + Glucose_ B	Hematite + Glucose_ A	Hematite + Glucose_ B	Glucose_ A	Glucose_ B	Unamended_A	Unamended_B
0	4 ºC	7.21	7.1	7.15	7.12	7.12	7.08	7.14	7.13
14		6.89	6.89	6.91	6.83	6.93	6.9	7.24	7.28
77		7	7.02	6.9	6.94	6.94	6.96	7.24	7.31
100		6.98	7.01	7.02	6.99	6.88	6.92	7.32	7.28
0	10 ºC	7.17	7.16	7.21	7.16	7.32	7.24	7.31	7.24
14		6.92	6.93	6.91	6.82	6.88	6.88	7.19	7.22
77		6.98	7.04	7.04	7.03	7.23	7.26	7.23	7.26
100		7.01	6.97	7.02	7	6.95	6.96	7.25	7.34
0	30 ºC	7.13	7.19	7.15	7.22	7.15	7.35	7.18	7.17
14		6.89	6.92	7.05	6.9	6.85	6.88	7.22	7.18
77		7.05	7.03	7.08	7.05	6.97	7.03	7.18	7.14
100		7.02	6.97	6.89	6.86	6.92	6.96	7.25	7.28

Data shows carbonate concentrations in the natural carbonate buffer system in the sediment is sufficient to keep the pH of the media stable over time as only slight changes were observed in incubations amended with glucose over time.

Table S3. Absolute numbers of sequence reads processed per sample after OTU classification. Archaea 16S rRNA reads in the bacteria sequences and vice versa was removed before arriving at absolute numbers.

Treatment	4 ºC			10 ºC			30 ºC		
	Time (days)	Bacteria	Archaea	Time (days)	Bacteria	Archaea	Time (days)	Bacteria	Archaea
day 0.a		28546	4318		25256	4180		11450	6591
day 0.b	0	19950	3646	0	26023	1891	0	19615	5083
day 0.c		13798	7386		27788	8592		17982	3385
G.a		28500	3077		33363	7242		26693	3489
G.b		17283	2392		32583	3222		17817	3348
G.c		13174	2550		27412	6563		16339	3175
MG.a		13931	1421		15186	4709		10184	2566
MG.b	21	24287	3118	90	21907	8333	23	26935	1492
MG.c		20235	4118		19129	4578		24041	3023
HG.a		19302	2809		17105	6143		23208	1789
HG.b		26092	3271		13894	7180		18273	1699
HG.c		19733	2227		26800	1781		21641	2466
G.a		57136	1429		41484	11515		25290	7277
G.b		61605	1336		53555	3319		52879	4740
G.c		98905	2255		40997	7809		29286	5558
MG.a		34489	3432		18151	4048		13546	5527
MG.b	83	32552	1883	200	34882	24628	50	8267	5549
MG.c		295843	2912		29285	7773		11893	6112
HG.a		50565	1243		20338	8554		13910	6807
HG.b		41664	1262		14696	11564		25312	4846
HG.c		45562	1826		42890	3132		18841	6589
G.a		24800	8057						
G.b		19682	3141						
G.c		18115	8980						
MG.a		13884	2791						
MG.b	782	6395	4769						
MG.c		11906	6057						
HG.a		12216	9376						
HG.b		19020	9437						
HG.c		18309	11681						

Absolute numbers used to determine relative abundance (%) of individual members of the community (see methods). MG represents incubations amended with magnetite and glucose; HG represents hematite and glucose amendments while G represents glucose amended incubation.

Table S4. Absolute numbers of sequence reads processed per sample after OTU classification in the BES incubations at 30 ºC. Day 0 treatments for the BES incubations are the same day 0 samples used for the 30 ºC incubations without BES (See Table S2).

Treatment	BES incubations at 30 ºC		
	Time (days)	Bacteria	Archaea
BG.a	23	40742	3650
BG.b		25851	1673
BG.c		21701	NA
BMG.a		42359	3794
BMG.b		30696	2072
BMG.c		35359	2053
BHG.a		21998	3427
BHG.b		35357	5001
BHG.c		32684	3132
BG.a	50	15660	1680
BG.b		7478	1815
BG.c		28052	3104
BMG.a		27804	3678
BMG.b		19719	2829
BMG.c		37051	3354
BHG.a		11310	5684
BHG.b		27417	4753
BHG.c		24534	3016

NA represents the BG.c archaea sample at day 23 that failed during sequencing preparation. The unavailability of that sample however had no effect on the overall result as the other two replicates looked similar and the archaea community remained undisturbed over time across all BES amended incubations.
